# Decreased cigarette smoking may partially explain the increased prevalence of antinuclear antibodies in the United States

**DOI:** 10.3389/fimmu.2025.1537043

**Published:** 2025-08-27

**Authors:** Gregg E. Dinse, Clarice R. Weinberg, Christine G. Parks, Caroll A. Co, Jessica T. Priest, Edward K. L. Chan, Frederick W. Miller

**Affiliations:** ^1^ Public Health & Scientific Research, DLH, LLC, Bethesda, MD, United States; ^2^ Biostatistics and Computational Biology Branch, National Institute of Environmental Health Sciences, National Institutes of Health, Research Triangle Park, NC, United States; ^3^ Epidemiology Branch, National Institute of Environmental Health Sciences, National Institutes of Health, Research Triangle Park, NC, United States; ^4^ Department of Oral Biology, University of Florida Health Science Center, Gainesville, FL, United States; ^5^ Clinical Research Branch, National Institute of Environmental Health Sciences, National Institutes of Health, Research Triangle Park, NC, United States

**Keywords:** antinuclear antibodies (ANA), autoimmune diseases, carbon monoxide (CO), cotinine, e-cigarettes, National Health and Nutrition Examination Survey (NHANES), tobacco smoking, vaping

## Abstract

**Introduction:**

Despite well-known harmful health effects of smoking, research supports an inverse association with some autoimmune diseases. High-titer antinuclear antibodies (ANA) are associated with autoimmune diseases, and ANA prevalence in the US increased between 1988 and 2012. Tobacco smoking decreased during those years while vaping of electronic cigarettes (e-cigarettes) increased after their introduction in 2007. Carbon monoxide (CO) may ameliorate autoimmunity, and e-cigarettes deliver much less CO than regular cigarettes. We explored interdependencies among ANA, smoking, and time.

**Methods:**

We analyzed cross-sectional data on ANA and the primary nicotine metabolite, cotinine, in 13,288 participants ≥12 years old from three time periods (1988-1991, 1999-2004, 2011-2012) of the US National Health and Nutrition Examination Survey. Smoking exposure (none, passive, active) was inferred from serum cotinine. We used logistic regression to analyze ANA prevalence, adjusted for sex, age, and race/ethnicity.

**Results:**

Over the study periods, ANA prevalence was highest (13.3-19.2%) for nonsmokers but non-trending; lower (11.1-15.5%) for “passive” smokers but steadily increasing; and even lower for active smokers but increasing from 7.4% in 1999–2004 to 13.3% in 2011-2012. The increases in ANA among passive and active smokers were mainly in adolescents (ages 12–19 years). Smokers had reduced odds of ANA in 1999-2004, with an odds ratio (OR) of 0.65 and a 95% confidence interval (CI) of 0.45-0.93, but this association was weaker in 1988-1991 (OR=0.80; 95% CI:0.52-1.22) and 2011-2012 (OR=0.82; 95% CI:0.56-1.21).

**Discussion:**

Although smoking causes harmful health effects, ANA data are consistent with smoking playing a role in decreasing autoimmunity. Recent vaping among adolescents may partially explain their large increase in ANA prevalence. The inverse ANA association with smoking strengthened between 1988-1991 and 1999-2004 but then weakened by 2011-2012. The initial strengthening was potentially because nonsmokers were exposed to progressively less CO (and/or other components of secondhand smoke), due to tightened smoking restrictions, while the potential nicotine-associated protection against ANA may have weakened after e-cigarettes became a source. Smoking should not be recommended given its negative health impacts. However, further studies could elucidate new mechanisms, perhaps involving components of tobacco smoke or vaping, possibly enabling development of novel preventative or treatment measures.

## 1 Introduction

High-titer antinuclear antibodies (ANA) are biomarkers associated with many autoimmune diseases (1–6), some of which have increased in incidence over recent decades for unknown reasons. Previously, based on data from the US National Health and Nutrition Examination Survey (NHANES), we reported an increasing ANA time trend (7) and investigated possible ANA associations with 253 xenobiotics (8). Our initial goal was to explore whether temporal changes in the levels of any xenobiotics associated with ANA could help explain the increase in ANA prevalence over time. However, many xenobiotics were evaluated at only one point in time or had mostly undetectable levels. We ultimately focused on serum cotinine, which was measured in nearly all participants.

Smoking tobacco is a major cause of preventable deaths, illnesses, and health care costs worldwide (9, 10), but despite overwhelming evidence of harmful effects of smoking in general, smoking has appeared to be inversely associated with ANA (7). Cotinine has often been used as a biomarker for tobacco smoke exposure (11–13), and as the primary metabolite of nicotine, cotinine has long been regarded as the most reliable indicator of active and passive exposure to tobacco smoke (11, 14). However, cotinine can also signal other nicotine exposures such as nicotine gum, chewing tobacco, snuff, and snus. Recently, an increasingly popular nicotine-delivering alternative to regular cigarettes, electronic cigarettes (e-cigarettes), has expanded the opportunities for smokeless exposure to nicotine (15).

In this article, we explore whether the decrease in cigarette smoking over the past few decades (16, 17) could plausibly account for some of the increase in ANA. We assess associations seen in the large NHANES database, some of which were observed previously (7), and postulate a potentially protective (or immunosuppressive) effect of carbon monoxide (CO) that might help explain the apparent inverse correlation between cigarette smoking and ANA.

The effects of smoking and CO on autoimmune diseases depend on individual variability, exposure levels, and the disease in question. Perricone et al. (18) discuss numerous studies of the relationship between smoking and autoimmune diseases. While smoking is a risk factor for many autoimmune diseases, smoking appears to have a protective effect for others, including ulcerative colitis, celiac disease, Behcet’s disease, type 1 diabetes, and autoimmune hypothyroidism. Epidemiologic studies have suggested that smoking may protect against ulcerative colitis (19–21), Behcet’s disease (21), autoimmune hypothyroidism (22–24), and Sjogren’s syndrome (19, 20), and that CO may protect against discoid lupus erythematosus (25). Rodent studies have suggested that CO may have therapeutic effects for various autoimmune diseases, including multiple sclerosis (26, 27), collagen-induced arthritis (28), systemic lupus erythematosus (29), type 1 diabetes (30), uveitis (31), and autoimmune hepatitis (32).

Starting early this century, many smokers began using e-cigarettes, either in addition to or instead of regular cigarettes (15, 33–36). Among 116 adult e-cigarette users in one study (37), 68% self-reported as current smokers, 24% as former smokers, and 8% as never smokers. The use of e-cigarettes doubled between 2010 and 2013 among US adults, with over 20 million having tried them (34), and the use among high school students increased from 1.5% in 2011 to 16% in 2015 (15). E-cigarettes supply nicotine and thus cotinine (15, 38) but produce much less CO than regular cigarettes (39, 40). We hypothesize that a decrease in CO and/or other possibly “protective” smoking byproducts, either due to quitting all forms of smoking or switching fully or partially from regular cigarettes to e-cigarettes (or other nicotine delivery systems, such as chewing tobacco, snuff, or snus), may have contributed to the recent increase in ANA in the US.

## 2 Subjects and methods

### 2.1 Study participants

Data on ANA were available for 13,519 participants from five NHANES cycles: 1988-1991, 1999-2000, 2001-2002, 2003-2004, and 2011-2012. The NHANES sampled representative members of the noninstitutionalized civilian US population and provided sampling weights to adjust for selection probabilities and nonresponse (41), which enables inference that generalizes to most of the US population. All participants signed informed consent documents and completed questionnaires, and most were physically examined and provided blood and urine specimens. Available data included demographic characteristics, health covariates, measured factors, and constructed variables. The NHANES protocol was approved by the Human Subjects Institutional Review Board of the US Centers for Disease Control and Prevention (CDC).

### 2.2 ANA assessment

All serum samples were evaluated for ANA in the laboratory of Dr. Edward K.L. Chan between 2016 and 2017 by indirect immunofluorescence at a 1:80 dilution using the NOVA Lite HEp-2 ANA slide with DAPI kit (Inova Diagnostics, San Diego, California, USA), with a highly specific fluorescein isothiocyanate-conjugated secondary antibody (goat anti-human IgG). Immunofluorescence staining intensities were graded 0–4 compared to standard references (42). Grades 1–4 were considered positive for ANA and grade 0 was considered negative. For more assay details see Dinse et al. (7).

### 2.3 Data on ANA, cotinine, and smoking

For cost and other practical reasons, ANA were only assayed in a subset of participants ≥12 years old in each of the five cycles. The ANA subsamples from 1999-2000, 2001-2002, and 2003–2004 were each roughly one-third the size of those from 1988–1991 and 2011-2012. Thus, as in our earlier studies (7, 8), we combined the three middle cycles to create three time periods with similar sample sizes: 1988-1991 (N=4,727), 1999-2004 (N=4,527), and 2011-2012 (N=4,265). As before, we focused on these three periods rather than the five cycles.

All analyses were restricted to the 13,519 participants with ANA data. The CDC adjusted the sampling weights to account for analyzing this ANA subsample. Data were available on cotinine, and thus smoking exposure as defined by cotinine concentration, for 13,288 participants; on self-reported smoking history for 12,278 participants; and on both smoking exposure and smoking history for 12,063 participants. **Supplementary Table S1** shows the numbers of participants in each time period (and overall) with data on ANA, smoking exposure, and smoking history. Throughout this article, “cotinine” refers to serum cotinine and not urinary cotinine.

### 2.4 Model variables

The ANA outcome variable was a binary indicator of ANA positivity/negativity. Cotinine concentration (ng/mL) was a quantitative variable and was used to classify smoking exposure as none (≤0.05), passive (>0.05 to 10), or active (>10), as recommended by the CDC and the US Environmental Protection Agency (EPA) (13), though a sensitivity analysis applied a more recent recommendation of >3 ng/mL for defining active smoking exposure. Combining the first two exposure categories produced an indicator of smoking status: nonsmoker (none or passive exposure) versus smoker (active exposure). Smoking history was based on questionnaire data, with individuals self-reporting as never, former, or current smokers.

Except where otherwise noted, our primary analyses adjusted for sex, age, race/ethnicity, and the survey design variables (i.e., strata, clusters, and weights proportional to the inverse probability of sampling), each of which was available for all participants. Age was measured in years and categorized by decade (12-19, 20-29,…, 70-79, ≥80), though sensitivity analyses explored the use of fewer age categories, a quantitative age variable, or a restricted cubic spline in age. Self-reported race/ethnicity was categorized as non-Hispanic White, non-Hispanic Black, Mexican American, or Other. Secondary analyses adjusted for body mass index (BMI), alcohol intake, poverty income ratio (PIR), and education, as defined previously (42). Secondary analyses also investigated CO content in cigarettes, pack-years of smoking, lifetime years of smoking, and years since former smokers quit smoking, though these data were very limited.

### 2.5 Statistical analysis

When analyzing ANA prevalence, we used logistic regression models to allow the probability of ANA positivity to depend on explanatory variables. All models adjusted for the survey design variables. The basic model for estimating overall ANA prevalence and its 95% confidence interval (CI) did not include adjustment covariates, but we did include a categorical covariate for period when estimating ANA prevalence in each of the three time periods. When assessing ANA time trends, we adjusted for sex, age, and race/ethnicity and calculated an ANA prevalence odds ratio (OR) and its 95% CI for each period relative to the first period. The statistical significance of an ANA time trend was evaluated by replacing the categorical period covariate with a quantitative time covariate and then inspecting its p-value, where time was defined as the number of years between the midpoints of the participant’s period and the first period.

When analyzing the cotinine data, we calculated the geometric mean cotinine concentration for each time period. We also derived a trend line by using linear regression to model individual log-transformed cotinine concentration as a function of the number of years between the midpoints of the participant’s period and the first period. Any concentration below the limit of detection (LOD) was replaced by an imputed value of LOD/ $\sqrt{2}$ (43, 44). The cotinine LOD was initially 0.05 ng/mL but was lowered to 0.015 ng/mL during the second period due to an improvement in the assay; the corresponding imputed values were 0.035 and 0.011 ng/mL. We also evaluated mean cotinine concentrations over time (and estimated trend lines) within subgroups of self-reported never, former, and current smokers, and we used kernel density plots to assess the full cotinine concentration distribution for each smoking-history subgroup and time period.

When analyzing smoking time trends, we used logistic regression to estimate the prevalence of smokers in each time period. Overall prevalence estimates were adjusted for the survey design variables but not for any covariates. Also, after further adjusting for sex, age, and race/ethnicity, we estimated a prevalence OR (and a 95% CI) for each period relative to the first period.

When investigating the relationship between ANA and smoking, we performed logistic regression analyses similar to those described above for ANA prevalence. First, we stratified by smoking and analyzed ANA prevalence and time trends separately in each stratum. Second, we stratified by both age and smoking to see whether the ANA association with smoking depended on age. Third, we added a smoking covariate (instead of stratifying) and assessed whether that smoking covariate affected the ANA association with time or whether removing the period covariate altered the ANA association with smoking. Fourth, we also added a smoking-by-period interaction to evaluate whether smoking modified the ANA time trend. Fifth, we stratified by period and compared ANA prevalence for smokers versus nonsmokers to gauge how the ANA association with smoking changed over time.

Finally, we conducted sensitivity analyses to assess whether our results changed when using an alternative age covariate (fewer categories, quantitative, or restricted cubic spline) or when only considering adults (ages ≥20 years). We also explored the use of a more recent recommendation of >3 ng/mL for the cotinine cutpoint when defining active smoking exposure. In addition, we investigated several other covariates (BMI, alcohol intake, PIR, and education) and the limited data on cigarette CO content, pack-years, years of smoking, and years since quitting.

All analyses were performed with SAS software (version 9.4, SAS Institute, Cary, NC) and accounted for the survey design variables by using special survey procedures. Domain statements were used to properly handle the sampling weights in subgroup analyses. Variance estimates for the 95% CIs were obtained using the Taylor series method. Reported p-values were 2-sided. All plots were constructed in SAS except the kernel density plot, which was created in R (version 4.4.0, R Foundation, Vienna, Austria).

## 3 Results

### 3.1 ANA time trend

The prevalence of ANA rose over the 25-year span for which NHANES data on ANA were available, with most of the increase occurring between the second and third time periods. Accounting only for time period and the survey design variables, the weighted estimates of ANA prevalence were 11.0% (95% CI: 9.7-12.5%) in Period 1 (1988–1991), 11.4% (95% CI: 10.2-12.8%) in Period 2 (1999-2004), and 16.1% (95% CI: 14.5-17.9%) in Period 3 (2011-2012). These overall estimates, along with sample sizes and numbers of ANA-positive participants, are shown in the last row of **Table 1**. Relative to Period 1 and after further adjustment for sex, age, and race/ethnicity, the ANA prevalence OR was 1.02 (95% CI: 0.84-1.24) for Period 2 and 1.49 (95% CI: 1.23-1.82) for Period 3 (**Table 2**), and there was strong statistical evidence of a positive trend in ANA prevalence over time (p=0.0001). We reported these results earlier (7), with slight discrepancies due to minor differences in analysis, but repeat them here for context.

**Table 1:** Sample sizes, ANA-positive counts, and ANA prevalence estimates by time period and smoking exposure.

Smoking	Period 1: 1988-1991		Period 2: 1999-2004		Period 3: 2011-2012		All Periods Combined	
Exposure ^a^	N+/N	Prev (95% CI) ^b^	N+/N	Prev (95% CI) ^b^	N+/N	Prev (95% CI) ^b^	N+/N	Prev (95% CI) ^b^
None	93/429	19.2 (13.6-26.3)	264/1,884	13.3 (11.3-15.7)	401/2,379	17.4 (14.7-20.4)	758/4,692	16.0 (14.2-18.0)
Passive	343/2,739	11.1 (9.6-12.8)	190/1,581	12.7 (10.5-15.4)	141/1,001	15.5 (13.2-18.1)	674/5,321	12.5 (11.3-13.7)
Active	168/1,357	8.6 (6.4-11.4)	89/1,034	7.4 (5.6- 9.7)	127/884	13.3 (11.0-15.9)	384/3,275	9.5 (8.2-10.9)
Total	643/4,727	11.0 (9.7-12.5)	545/4,527	11.4 (10.2-12.8)	669/4,265	16.1 (14.5-17.9)	1,857/13,519	13.0 (12.1-13.9)

ANA, antinuclear antibodies; CI, confidence interval; LOD, limit of detection; N, total number of participants (sample size); N+, number of ANA-positive participants; Prev, ANA prevalence (as a percent).

^a^Smoking exposure categories were based on serum cotinine concentrations (None, ≤0.05 ng/mL; Passive, >0.05 to 10 ng/mL; and Active, >10 ng/mL). Due to a technical improvement in the cotinine assay, the cotinine LOD decreased from 0.05 to 0.015 ng/mL during Period 2. The number of participants with a missing cotinine value also decreased over time from 202 in Period 1 to 28 in Period 2, and then to 1 in Period 3.

^b^ANA prevalence was estimated under two logistic regression models for ANA positivity (yes/no), adjusted for the survey design variables (sampling weights, strata, and clusters). One model included only an intercept, which produced an overall estimate for all time periods combined. The other model included a categorical covariate for time period, which produced a separate estimate for each period. Both models were applied initially to all participants with data on ANA regardless of data on smoking exposure (Total) and then within subgroups with data on both ANA and smoking exposure (None, Passive, and Active). The subgroup counts sum to less than the total sample size because some participants were missing data on smoking exposure (i.e., cotinine).

**Table 2:** Covariate-adjusted assessments of ANA time trends by smoking exposure.

Smoking	ANA Prevalence Odds Ratio for Time Period (95% CI) ^b^			Time Trend
Exposure ^a^	Period 1: 1988-1991	Period 2: 1999-2004	Period 3: 2011-2012	p-value ^b^
None	1.00 (reference)	0.70 (0.45-1.09)	0.99 (0.62-1.57)	0.2139
Passive	1.00 (reference)	1.28 (0.96-1.69)	1.72 (1.33-2.22)	0.0001
Active	1.00 (reference)	0.81 (0.53-1.23)	1.45 (1.01-2.08)	0.0661
Total	1.00 (reference)	1.02 (0.84-1.24)	1.49 (1.23-1.82)	0.0001

ANA, antinuclear antibodies; CI, confidence interval.

^a^Smoking exposure categories were based on serum cotinine concentrations (None, ≤0.05 ng/mL; Passive, >0.05 to 10 ng/mL; and Active, >10 ng/mL).

^b^ANA time trend assessments were based on two logistic regression models for ANA positivity (yes/no). Each model adjusted for the survey design variables (sampling weights, strata, and clusters) and categorical covariates for sex, age, and race/ethnicity. One model added a categorical covariate for time period, which allowed estimates of the ANA prevalence odds ratio for each period relative to the first period. The other model instead added a continuous covariate for time, as measured by the number of years between period midpoints relative to the first period, and produced a p-value from a t-test to assess a linear ANA time trend. Both models were applied initially to all participants with data on ANA regardless of data on smoking exposure (Total) and then within subgroups with data on both ANA and smoking exposure (None, Passive, and Active).

### 3.2 Cotinine time trend

**Figure 1:**
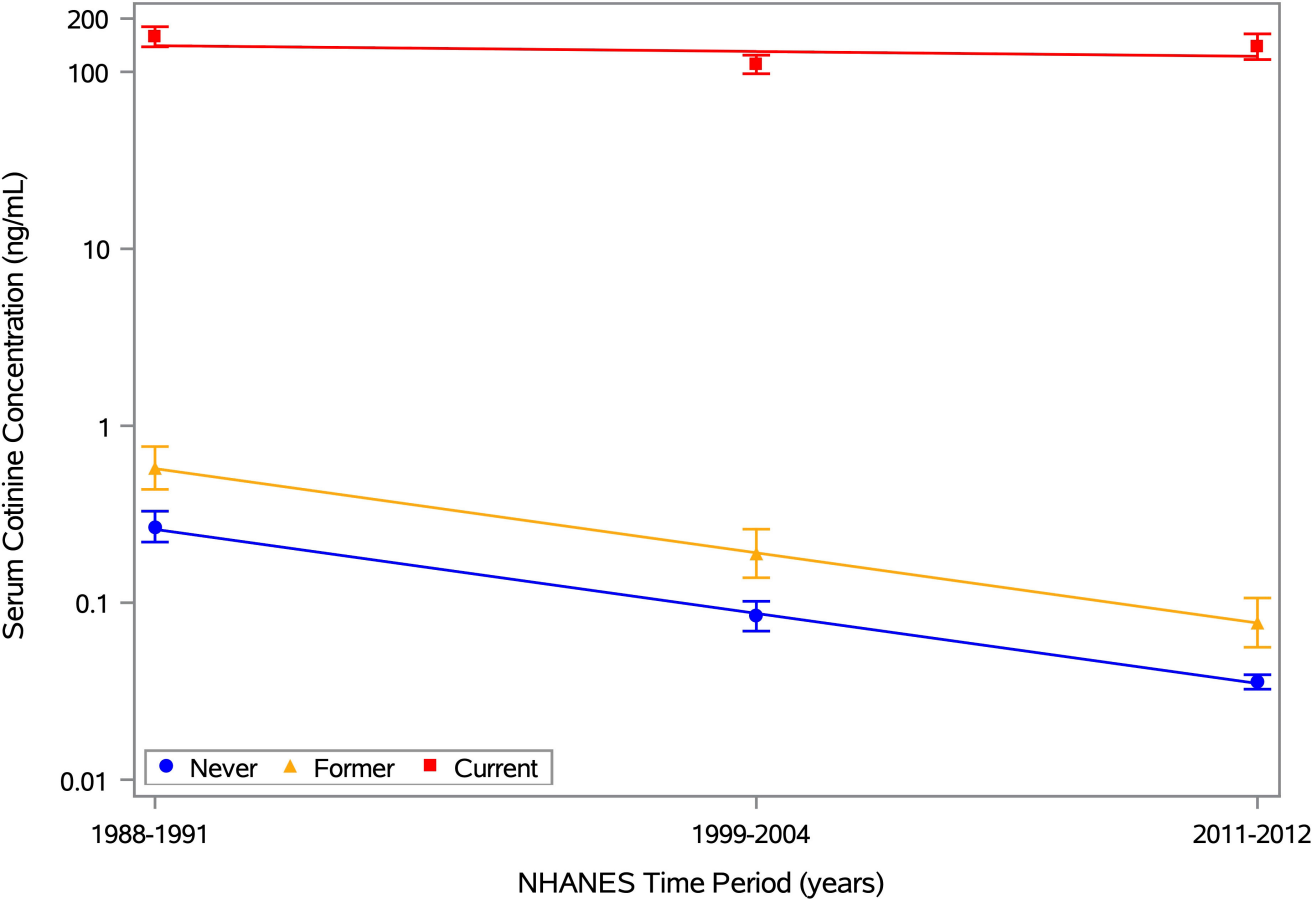
Mean serum cotinine concentration by time period and smoking history. Estimates of the geometric mean serum cotinine concentration and its 95% CI are plotted for each of 3 time periods (1988-1991, 1999-2004, and 2011-2012), along with the best-fitting trend line. Separate estimates are shown for self-reported never, former, and current smokers, based on the 12,063 NHANES participants aged ≥12 years with data on ANA, serum cotinine, and smoking history. The means for never, former, and current smokers are depicted by blue circles, yellow triangles, and red squares, respectively, with the same colors used for the 95% CI error bars and trend lines. Any concentration below the limit of detection (LOD) was replaced by an imputed value equal to LOD/ $\sqrt{2}$ . The horizontal axis is linear in time, defined as the number of years between the midpoints of the participant’s period and the first period, and the vertical axis is logarithmic in serum cotinine concentration (ng/mL).

**Supplementary Figure S1** shows the geometric mean cotinine concentration and its 95% CI for each period, along with the best-fitting trend line. There was strong statistical evidence (p<0.0001) of a steady decrease over time. When stratified by self-reported smoking history, the mean cotinine levels ranged from 0.04 to 0.27 ng/mL for never smokers, 0.08 to 0.59 ng/mL for former smokers, and 104.2 to 158.5 ng/mL for current smokers (top half of **Supplementary Table S2**). The best-fitting trend line had a negative slope in all three subgroups but was steeper for never and former smokers than for current smokers (**Figure 1**). Also, the decrease over time was statistically significant for both never and former smokers (p<0.0001), but not for current smokers (p=0.08). Thus, on average, current smokers had cotinine levels that were high and fairly constant over time, while former and never smokers had levels that were low and decreasing, likely due to steady reductions in secondhand smoke exposure. Similar results were obtained when excluding participants under age 20 years (bottom half of **Supplementary Table S2**) to account for smoking history data being available for different age ranges across time periods (≥17 years in Period 1, ≥12 years in Period 2, and ≥20 years in Period 3).

**Figure 2:**
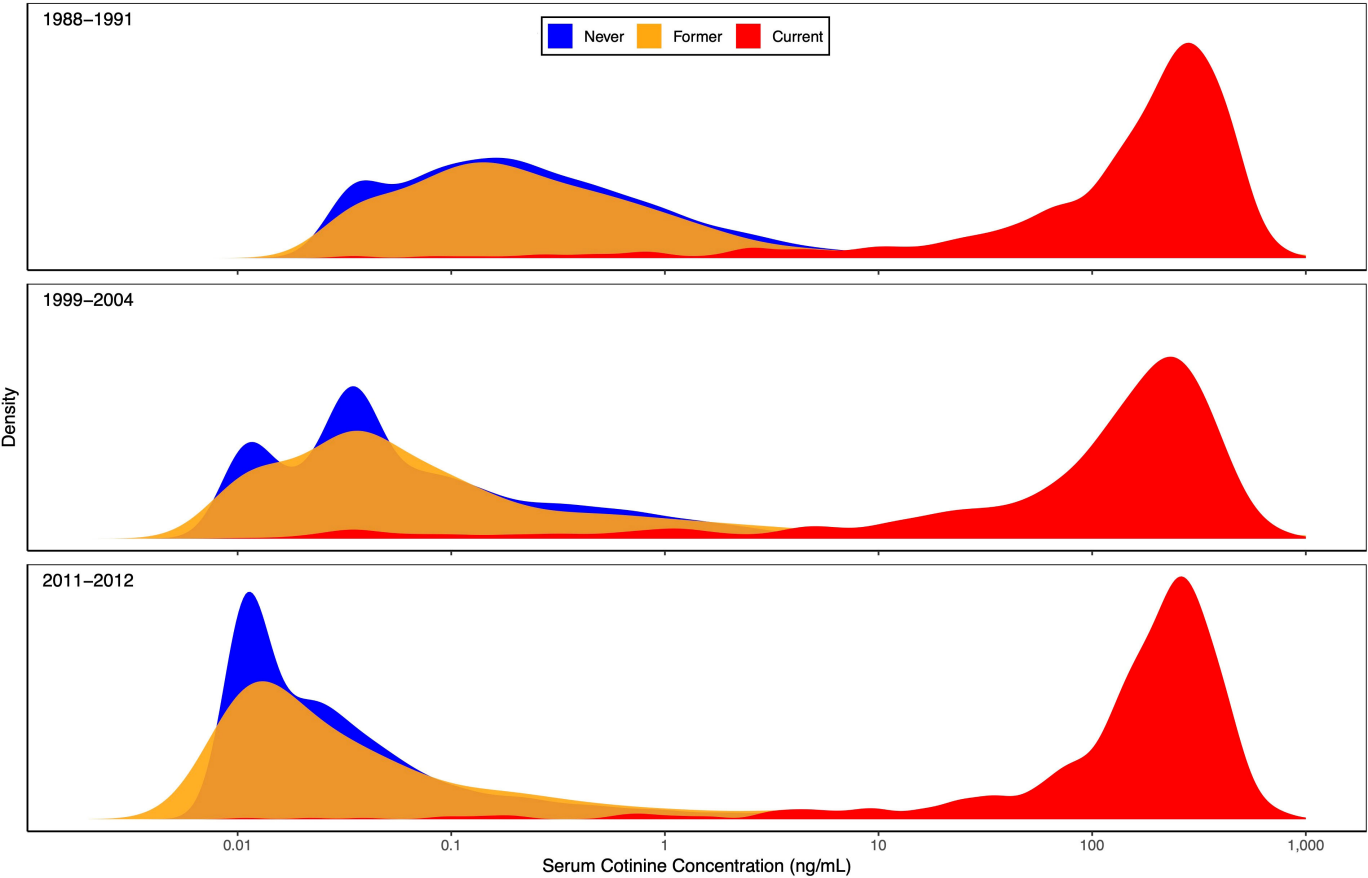
Serum cotinine concentration distribution by time period and smoking history. Kernel density estimates of the entire serum cotinine concentration distribution are plotted for each of 3 time periods (1988-1991, 1999-2004, and 2011-2012). Separate estimates are shown for self-reported never (blue), former (yellow), and current (red) smokers, based on the 12,063 NHANES participants aged ≥12 years with data on ANA, serum cotinine, and smoking history. Any concentration below the limit of detection (LOD) was replaced by an imputed value equal to LOD/ $\sqrt{2}$ . The horizontal axis is logarithmic in serum cotinine concentration (ng/mL).

Rather than focusing on means, **Figure 2** displays kernel density estimates of the entire distribution of cotinine concentrations by time period and smoking history. These plots clearly show the differences in cotinine levels for never and former smokers (low) versus current smokers (high), as well as the consistency over time for current smokers. The cotinine distributions for never and former smokers were less consistent, with a notable shift toward lower values as time progressed. Much of this shift was likely due to many never and former smokers having cotinine levels below the LOD, which decreased from 0.05 to 0.015 ng/mL in the second time period. Nondetectable levels were replaced by imputed values of 0.035 and 0.011 ng/mL, respectively, which match well with the peaks of the period-specific cotinine distributions for never smokers. The cotinine distributions were more spread out for former smokers than for never smokers, perhaps due to a larger proportion of former smokers interacting with a current smoker.

### 3.3 Smoking time trend

Cigarette smoking in the US has decreased for a half-century (16, 17). We confirmed this downward trend in the NHANES data by examining the proportions of active smokers (defined by cotinine levels) and current smokers (based on self-reports), both of which clearly decreased over time. Unadjusted period-specific estimates of smoking prevalence for both classifications demonstrated similar decreases among all participants and among adults only (**Supplementary Table S3**), as did covariate-adjusted estimates of the smoking prevalence ORs for time period (**Supplementary Table S4**).

### 3.4 ANA time trends by smoking exposure

Estimates of ANA prevalence exhibited different temporal patterns in the three smoking exposure subgroups. For individuals with no exposure, these estimates were highest but did not show a clear trend; for passive exposure, they were intermediate and increased steadily across all periods from 11.1% (95% CI: 9.6-12.8%) to 12.7% (95% CI: 10.5-15.4%) to 15.5% (95% CI: 13.2-18.1%); and for active exposure, they were lowest and initially flat but then rose markedly from 7.4% (95% CI: 5.6-9.7%) in Period 2 to 13.3% (95% CI: 11.0-15.9%) in Period 3 (**Table 1**). Covariate-adjusted estimates of the ANA prevalence OR for Period 3 relative to Period 1 were 0.99 (95% CI: 0.62-1.57) for no exposure, 1.72 (95% CI: 1.33-2.22) for passive exposure, and 1.45 (95% CI: 1.01-2.08) for active exposure (**Table 2**). When assessing a linear trend in ANA prevalence across all three periods, the p-values for the three exposure subgroups were 0.2139, 0.0001, and 0.0661, respectively (**Table 2**). We reported similar estimates previously (7), but with smoking exposure categories defined by slightly different cutpoints for cotinine concentration.

To investigate whether age modified the association between smoking and temporal patterns of ANA, in addition to stratifying by smoking exposure, we further stratified by three age groups (12-19, 20-49, and ≥50 years) instead of including a categorical covariate for age. This approach essentially allowed for interactions between age and the covariates (sex, race/ethnicity, and time period). Despite the larger number of subgroups leading to smaller counts within each, there was statistical evidence that the observed increase in ANA prevalence over time was associated mainly with 12–19 year-olds who were passive (p=0.005) or active (p=0.003) smokers (**Table 3**). Among adolescents and relative to Period 1, the ORs and 95% CIs for passive smokers were 1.63 (0.83-3.23) in Period 2 and 2.64 (1.37-5.08) in Period 3, and for active smokers they were 3.01 (0.53-17.3) in Period 2 and 9.92 (2.20-44.7) in Period 3. The wide CIs are indicative of the small counts, but the ORs are large, especially for adolescents who were active smokers (which would have included vapers), for whom the odds of being ANA positive were roughly 10 times greater in Period 3 compared with Period 1. The differences across age categories, based on assessing an interaction between age group and time period, were statistically significant (p=0.009).

**Table 3:** Covariate-adjusted assessments of ANA time trends by smoking exposure and age group.

Smoking	ANA Prevalence Odds Ratio for Time Period (95% CI) ^b^				Time Trend
Exposure ^a^	Period 1: 1988-1991	Period 2: 1999-2004	Period 3: 2011-2012		p-value ^b^
Age Group 1: 12–19 years old					
None	1.00 (reference)	3.64 (0.99-13.4)	2.84 (0.75-10.8)	0.5002	
Passive	1.00 (reference)	1.63 (0.83-3.23)	2.64 (1.37-5.08)	0.0047	
Active	1.00 (reference)	3.01 (0.53-17.3)	9.92 (2.20-44.7)	0.0032	
Age Group 2: 20–49 years old					
None	1.00 (reference)	0.39 (0.21-0.72)	0.58 (0.32-1.06)	0.9447	
Passive	1.00 (reference)	1.09 (0.70-1.70)	1.53 (0.91-2.60)	0.1435	
Active	1.00 (reference)	0.73 (0.43-1.24)	1.28 (0.82-2.00)	0.4474	
Age Group 3: ≥50 years old					
None	1.00 (reference)	0.87 (0.47-1.60)	1.29 (0.70-2.36)	0.1303	
Passive	1.00 (reference)	1.37 (0.91-2.06)	1.41 (0.96-2.06)	0.0452	
Active	1.00 (reference)	0.85 (0.48-1.47)	1.43 (0.78-2.63)	0.2353	

ANA, antinuclear antibodies; CI, confidence interval.

a Smoking exposure categories were based on serum cotinine concentrations (None, ≤0.05 ng/mL; Passive, >0.05 to 10 ng/mL; and Active, >10 ng/mL).

b ANA time trend assessments were based on two logistic regression models for ANA positivity (yes/no). Both models stratified by smoking exposure and age group, and both adjusted for the survey design variables (sampling weights, strata, and clusters) and categorical covariates for sex and race/ethnicity. One model added a categorical covariate for time period, which allowed estimates of the ANA prevalence odds ratio for each period relative to the first period. The other model instead added a continuous covariate for time, as measured by the number of years between period midpoints relative to the first period, and produced a p-value from a t-test to assess a linear ANA time trend.

### 3.5 ANA associations with smoking by time period

**Figure 3:**
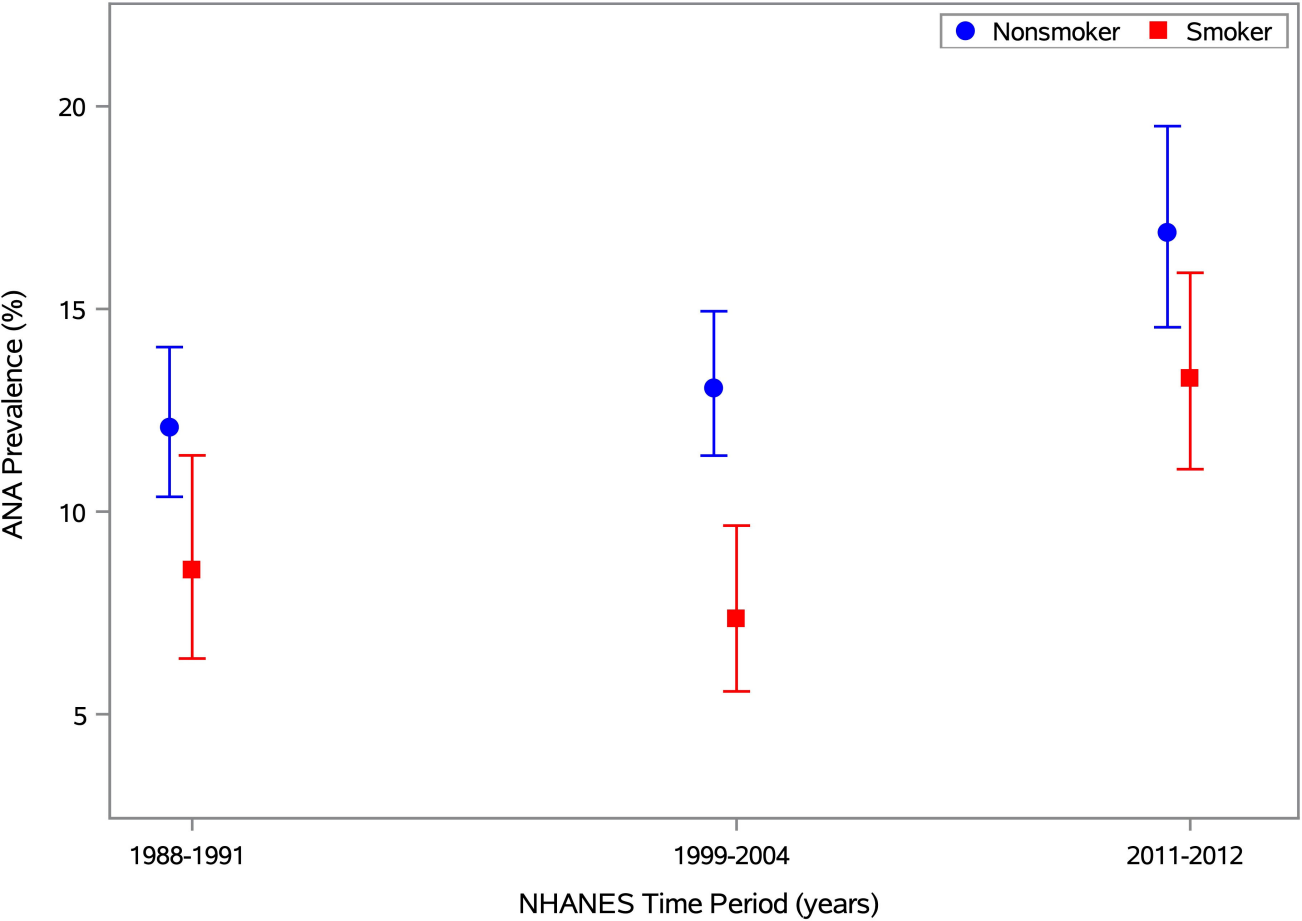
ANA prevalence by time period and smoking status. Estimates of ANA prevalence and its 95% CI are plotted for smokers and nonsmokers in Period 1 (1988-1991), Period 2 (1999-2004), and Period 3 (2011-2012), based on the 13,288 NHANES participants aged ≥12 years with data on both ANA and smoking status (i.e., serum cotinine). The prevalence estimates for nonsmokers and smokers are shown by blue circles and red squares, respectively, with the same colors used for the 95% CI error bars. Separately for each smoking status, the prevalence estimates and 95% CIs were derived from a logistic regression model for ANA positivity, adjusted for the survey-design variables and a categorical covariate for time period. The horizontal axis is linear in time, defined as the number of years between the midpoints of the participant’s period and the first period, and the vertical axis is linear in ANA prevalence (as a percentage).

In an alternative covariate-adjusted analysis, we focused on smoking status and assessed the odds of ANA positivity for smokers relative to nonsmokers (**Table 4**). Overall, smokers were less likely to have ANA than nonsmokers (OR=0.73; 95% CI: 0.58-0.92; p=0.007). When stratified by time period, the ANA prevalence ORs for smoking status varied in magnitude and statistical significance but not in direction. The odds of having ANA were significantly lower for smokers than nonsmokers in Period 2 (OR=0.65; 95% CI: 0.45-0.93; p=0.020), but that inverse association was weaker and not statistically significant in Period 1 (OR=0.80; 95% CI: 0.52-1.22; p=0.297) and Period 3 (OR=0.82; 95% CI: 0.56-1.21; p=0.310). This nonmonotonic temporal pattern is illustrated in **Figure 3**, where ANA prevalence estimates are smaller for smokers than nonsmokers in all three time periods, but the difference is much greater in Period 2 than in Periods 1 and 3.

**Table 4:** Covariate-adjusted assessments of ANA associations with smoking status by time period.

Time Period	ANA Prevalence Odds Ratio for Smoking Status (95% CI) ^a^		p-value ^a^
	Nonsmoker	Smoker	
Period 1: 1988-1991	1.00 (reference)	0.80 (0.52-1.22)	0.297
Period 2: 1999-2004	1.00 (reference)	0.65 (0.45-0.93)	0.020
Period 3: 2011-2012	1.00 (reference)	0.82 (0.56-1.21)	0.310
All Periods Combined	1.00 (reference)	0.73 (0.58-0.92)	0.007

ANA, antinuclear antibodies; CI, confidence interval.

a Assessments of the association between ANA and smoking status were based on a logistic regression model for ANA positivity (yes/no) that adjusted for the survey design variables (sampling weights, strata, and clusters) and categorical covariates for sex, age, and race/ethnicity. The model also included a categorical covariate for smoking status, as defined by serum cotinine concentrations (Nonsmoker, ≤10 ng/mL; Smoker, >10 ng/mL), which allowed estimates of the ANA prevalence odds ratio for smokers relative to nonsmokers. The model was applied separately for each time period and also for all periods combined. The p-value for assessing statistical significance was based on a t-test.

### 3.6 Additional analyses

We performed several sensitivity analyses by adding covariates to a base model that was adjusted for sex, age, race/ethnicity, and time period (**Supplementary Table S5**). Rather than stratifying by smoking exposure, including it as a categorical covariate led to the same basic pattern of ANA prevalence not changing much between Periods 1 and 2, followed by a marked increase in Period 3. When we also added a smoking-by-period interaction, the main effects of both smoking and period were statistically significant, but the interaction was not. On the other hand, excluding time period significantly worsened the model fit (p<0.0001), suggesting that calendar time was important and that smoking on its own could not fully explain the observed ANA differences.

We also performed secondary analyses that accounted for BMI, which had been shown previously to modify ANA time trends (7). Adding a 3-level categorical covariate for BMI (underweight/healthy, <25; overweight, 25 to <30; or obese, ≥30) to the base model did not change the ANA time trends, nor did also adding a BMI interaction with any factor in the base model or in an expanded model that also included a covariate for smoking exposure (**Supplementary Table S5**). Similarly, the original ANA time trends within smoking exposure subgroups (as shown in **Table 2**) did not change much when augmenting the base model with covariates for a BMI main effect and a BMI-by-age interaction (**Supplementary Table S6**).

Replacing the 8-category age covariate with a 3-category age covariate, a quantitative age covariate, or a restricted cubic spline in age did not alter the basic pattern of ANA prevalence being relatively flat between Periods 1 and 2, and then increasing substantially in Period 3 (**Supplementary Table S5**). Similarly, that basic ANA pattern also remained consistent when other covariates were added to the base model, such as an individual main effect for alcohol intake, PIR, or education; those same main effects plus a main effect for BMI; and those same main effects plus both a main effect for BMI and an interaction between BMI and each of those other covariates.

Data were available on the CO content in the brand of cigarettes used by each of 1,157 current smokers from Periods 2 and 3 aged ≥20 years. After adjusting for sex, age, race/ethnicity, and time period, there was mild evidence that ANA prevalence decreased as CO content per cigarette increased (OR=0.92; 95% CI: 0.85-1.00; p=0.042). We also multiplied CO content by average number of cigarettes smoked per day to estimate total CO, but the covariate-adjusted analysis showed no evidence of an association with ANA (p=0.65). In additional covariate-adjusted analyses of participants of all ages from all time periods, we found no evidence of an ANA association with pack-years among 4,795 ever (former or current) smokers (p=0.91), years of smoking among 5,047 ever smokers (p=0.18), or years since quitting among 2,738 former smokers (p=0.56). We also allowed for various pack-year threshold values, but no ANA associations with pack-years were significant.

## 4 Discussion

In summary, we assessed representative US data regarding ANA, time, smoking, CO and their interdependencies. **Table 5** lists several relevant concepts and results, including the following information. High-titer ANA are associated with autoimmune diseases (1–6). In the US, ANA increased from 1988 to 2012, primarily in the second half of that interval (7); both active and passive exposure to smoke from regular cigarettes decreased during those years (16, 17); and e-cigarette use rapidly increased after being introduced in 2007 (15, 33–36), especially among adolescents (ages 12–19 years). CO may protect against ANA and certain autoimmune diseases (18–20, 25–32). E-cigarettes deliver much less CO than regular cigarettes (39, 40).

**Table 5:** Concepts and results related to the hypothesis that decreased cigarette smoking may partially explain the increased prevalence of antinuclear antibodies in the United States.

1. High-titer antinuclear antibodies (ANA) are associated with some autoimmune diseases, and ANA prevalence estimates increased over time: a little between Period 1 (1988-1991) and Period 2 (1999-2004) and a lot between Period 2 and Period 3 (2011-2012).
2. Viewing ANA time trends by smoking exposure, there was no clear trend over time in ANA prevalence estimates for individuals with no exposure (negligible serum cotinine), a steady increase for individuals with passive exposure (low serum cotinine), and a flat-then-increasing trend for individuals with active exposure (high serum cotinine). The ANA time trends among passive and active smokers were associated mainly with 12–19 year-olds.
3. Viewing ANA associations with smoking by time period, the estimated odds of having ANA were less among active smokers (high serum cotinine) than nonsmokers (negligible or low serum cotinine) in all time periods, but only the difference in Period 2 was statistically significant.
4. Serum cotinine steadily decreased over time, primarily in self-reported never and former smokers, but not in self-reported current smokers.
5. Smoking of regular cigarettes and secondhand exposure to their smoke steadily decreased over time.
6. Vaping of electronic cigarettes (e-cigarettes) began after Period 2 (in 2007) and rapidly increased over time.
7. Both regular cigarettes and e-cigarettes deliver nicotine and hence produce cotinine, but e-cigarettes produce much less carbon monoxide (CO) than regular cigarettes.
8. Some studies suggest that low levels of CO may be protective against ANA and certain autoimmune diseases.
9. In summary, less smoking of regular cigarettes may have led to less low-level CO exposure and more ANA. The hypothesized explanation involving potential CO protection against ANA is consistent with the observed patterns of ANA prevalence estimates, the long-term decreases in secondhand smoke exposure, and the recent increases in vaping, especially among adolescents (12–19 years old).

Our general observation is that something related to smoking cigarettes appears to have been inversely associated with ANA and any potentially protective effect waned in the later time period, possibly because people were smoking less and vaping more, or because something else about smoking changed. In most cases, our use of the word “protective” refers to a statistical association and not a proven biological protection. We hypothesize that reduced CO from decreased exposure to cigarette smoke may account for some of the overall increase in ANA. This reduction in CO could have come from current smokers cutting back on their cigarette consumption (including some degree of switching to vaping), from former smokers who quit (and possibly switched to vaping), and from never or passive smokers being exposed to less secondhand smoke (due to regulations and social pressure). We also hypothesize that the rapid increase in e-cigarette use after 2007, especially among teenagers, may partially explain why the increase in ANA prevalence was larger during the latter half of the study years and why the increasing ANA time trend was the most pronounced in teenagers (7). Our two-part hypothesis is consistent with what is already known about ANA, smoking, and CO, as well as with the results from our analyses of the NHANES data. Specifically, we assessed how the ANA time trend depended on smoking exposure levels, including within age subgroups, and how the ANA association with smoking depended on calendar time. Both are described below.

The ANA time trends across the three smoking-exposure subgroups (as defined by serum cotinine level) are consistent with our hypothesis. Individuals with no smoking exposure had negligible cotinine levels and presumably were not affected by changes in vaping or secondhand smoke. Thus, we infer that their exposure to CO from cigarette smoke was minimal and, consistent with our hypothesis, their ANA prevalence showed no clear time trend. Individuals with passive exposure to smoke had detectable but relatively low cotinine levels, which means they would have been affected by changes in secondhand smoke but probably were not regular vapers. Hence, these individuals might have experienced a small but steady increase in ANA prevalence across all time periods, which we speculate could be due to the steady decrease in their low-level CO “protection” from decreasing secondhand smoke (and possibly also from reduced exposure via air pollution (https://www.epa.gov/air-trends/carbon-monoxide-trends)). Active smokers had high cotinine levels, which could result from either regular cigarettes or e-cigarettes, and would have been affected by changes in vaping but not secondhand smoke. Thus, these individuals presumably would not have had any change in potential CO protection or ANA prevalence between Periods 1 and 2, since vaping did not begin until 2007, but would have had a decrease in potential CO protection and, consistent with our hypothesis, a corresponding increase in ANA prevalence between Periods 2 and 3, as some of them took up vaping. Therefore, our hypothesis regarding potential smoking-associated CO protection from ANA is consistent with the possibility that the observed ANA patterns could be at least partially explained by the continued decrease in secondhand smoke exposure and the recent increase in vaping. In fact, when viewed by age group, the largest increase in ANA prevalence was between Periods 2 and 3 in teenagers who were active smokers, the timeframe and age group most associated with vaping.

The ANA associations with smoking seen across the three time periods are also consistent with our hypothesis and may relate to events that affected nonsmokers in the early years and smokers in the later years. Active smokers had significantly lower odds of having ANA than nonsmokers in Period 2, as would be expected if CO is protective, but this evident reduction was weaker (and not significant) in Periods 1 and 3. Between the first two periods, secondhand smoke exposure decreased (which would only affect nonsmokers) but vaping had not yet been introduced (which could only affect smokers who later started switching to e-cigarettes). All smokers had active smoking exposure, but nonsmokers were a mix of individuals with no exposure and passive exposure. The proportion of nonsmokers with passive exposure decreased over time, as presumably did their potential CO protection from secondhand smoke, and thus their ANA prevalence would have increased. However, neither potential CO protection nor ANA prevalence would have changed among smokers. Hence, the odds of having ANA for smokers versus nonsmokers would be smaller in Period 2 than in Period 1 (as we observed). Between Periods 2 and 3, secondhand smoke exposure again decreased (which would only affect nonsmokers) while vaping increased rapidly (which would mainly affect cotinine-identified active smokers). As described above, the level of potential CO protection from secondhand smoke among nonsmokers would have decreased, increasing their ANA prevalence. Concurrently, potential CO protection among active smokers (some of whom were vapers) would also have decreased due to increased vaping, and thus their ANA prevalence would have increased. The increase in ANA due to increased vaping among smokers could have more than offset the increase in ANA due to decreased secondhand smoke among nonsmokers, resulting in the ANA prevalences for smokers and nonsmokers to appear more similar in Period 3 than in Period 2 (as we observed).

Although we hypothesize that decreased CO and increased vaping may help explain both the changes in ANA time trends across smoking exposure levels and the changes in ANA associations with smoking across time periods, other factors may also have played a role. For example, cigarette smoke is composed of many chemicals with a wide array of effects on the body and we have an incomplete understanding of their immune impacts that could include both stimulatory and inhibitory elements that may vary from product to product (18, 45). Also, certain components of e-cigarettes, such as flavoring agents (46), may potentially increase the risk of developing ANA in users, and vaping may introduce additional chemical contaminants contributing to bystander health effects from secondhand exposure (47). In addition to vaping, there are other nicotine-delivering alternatives to regular cigarettes, including nicotine gum, chewing tobacco, snuff, and snus (48), that can have immune system effects (49). Another consideration is that some ANA subtypes may be more relevant than others. In a previous study (50), we found that time period and smoking exposure were more strongly associated with anti-dense fine speckled 70 autoantibodies than with total ANA. Miller (51) discussed a wide range of other potentially relevant factors such as elements of the environment, various lifestyles, and even climate change that could impact recent increases in autoimmunity and autoimmune diseases.

Our study had several strengths. The NHANES cohort with data on ANA was very large and spanned 25 years (1988–2012), with all ANA assays performed in the same laboratory, using the same evaluators, methods, and equipment. All statistical analyses were weighted to enable analytic results that generalize to the civilian noninstitutionalized US population ≥12 years old. Many of our analyses of ANA, cotinine, and smoking accounted for sex, age, and race/ethnicity as potential correlates or modifiers, and some analyses also adjusted for BMI, alcohol intake, PIR, or education.

On the other hand, our descriptive findings are subject to certain limitations. There may be concerns about the age of serum samples used for ANA assessment, some of which were nearly three decades old when assayed. However, there were no gross differences in appearance or behavior to suggest degradation, and antibodies are stable over time in frozen storage (52). Some NHANES data were obtained from questionnaires, such as smoking history, but self-reported nicotine product use has been shown to be valid (53). As vaping has increased, high cotinine levels have become less reliable for identifying persons who only smoke regular cigarettes (and thus are exposed to more CO). We considered using self-reported smoking history instead, but that information was often missing and it was not clear whether persons who replaced some or all of their regular cigarettes with e-cigarettes would classify themselves as former or current smokers. Also, we used 10 ng/mL of cotinine to distinguish passive and active smokers, as recommended by the CDC and EPA (13), but some researchers have suggested using a lower cutpoint, such as 3 ng/mL (54). However, our sensitivity analysis found that using the lower cotinine cutpoint had little effect on the results. No participant was followed longitudinally; thus, both cotinine and ANA were assessed cross-sectionally at only one point in time per participant, so measured cotinine levels may poorly reflect the levels when ANA developed. Reported associations, even if confirmed, may not correspond to causal effects. In fact, there could be reverse-causal effects if immune system or other changes associated with ANA influence smoking behavior or the metabolism of nicotine, cotinine, or other byproducts of smoking.

Perhaps the most serious deficiency in our data is the lack of direct information about e-cigarette use. At the time of our analyses, there were limited NHANES data on vaping in the 2013-2014, 2015-2016, and 2017–2018 cycles, but none in cycles with data on ANA. However, despite this absence of direct data, we might assume that most self-reported current smokers in Period 3 with a high cotinine level probably smoked regular cigarettes, whereas most self-reported former smokers with a high cotinine level had probably switched to e-cigarettes. The first group included 89/639 (13.9%) with ANA, while the second group included 18/85 (21.2%) with ANA, a difference that is consistent with our hypothesis of a potentially protective effect of CO derived from smoking regular cigarettes (and also consistent with an effect of something in e-cigarettes on ANA). Also, direct information on individual CO levels would have been helpful, though we found some evidence that lower ANA prevalence was associated with cigarette brands having higher CO content, which provides additional indirect support for our hypothesis.

In conclusion, cigarette smoking decreased over the past several decades and ANA prevalence increased, which we corroborated with analyses of NHANES data. However, the degree to which these two time trends might be causally related is unclear. Cotinine was used to infer exposure to cigarette smoke, and average levels steadily declined between 1988 and 2012 in the NHANES cohorts, with a downward-sloping straight line providing a good fit to log-transformed cotinine concentrations. The prevalence of ANA rose between 1988 and 2012, but this upward trend was not linear, showing a relatively small increase from 1988–1991 to 1999-2004, followed by a much larger increase from 1999–2004 to 2011-2012. The latter time interval coincides with the introduction of vaping, with many smokers replacing at least some of their regular cigarettes with e-cigarettes. That change might not have affected cotinine levels but should have reduced CO levels. We suggest that such a drop in CO levels potentially could be causally associated with the concurrent increase in ANA, as there is evidence that low levels of CO are protective against ANA and certain autoimmune diseases. However, while CO may be one factor in this process, one should keep in mind that there are many additional byproducts of smoking that possibly could play a role. Nonetheless, decreased smoking exposure (active and passive) across all study years could have contributed to a general increase in ANA, which could have been greatly supplemented in the later years by the rapid increase in vaping. Thus, smokers who reduced their use of regular cigarettes in favor of vaping may have lost some of the hypothesized protective effect afforded by CO, which could have increased their risk of developing autoimmunity.

We searched the literature for additional mechanisms and contributing factors that might help explain why decreased smoking could lead to increased ANA and found conflicting data on the complex mixtures that make up tobacco smoke and e-cigarette vapor. One parallel mechanism to CO is nicotine itself. Reduced cigarette smoking, if not replaced by other nicotine sources (48), would decrease the nicotine anti-inflammatory processes, which could then increase inflammation and ANA. For example, despite smoking being an established risk factor for rheumatoid arthritis (RA), several investigators have discussed a possible therapeutic effect of nicotine on RA (55–57). In the end, we concluded that exact mechanisms for why less smoking is associated with more ANA are unclear and further research is needed to identify the causes of the recent dramatic increases in ANA in the US. Hopefully, future studies will collect data on vaping history and CO biomarkers, which could provide direct evidence to assess our hypothesis.

In closing, given the many negative effects of smoking on increasing deaths, illnesses, and health care costs worldwide, we are certainly not recommending that smoking should be considered as an approach to prevent autoimmunity or autoimmune diseases. Rather, we believe that further studies in this area are needed as they may elucidate new mechanisms, perhaps involving certain components of tobacco smoke or e-cigarette vapor, that could allow for the development of novel preventative or treatment measures in the future.

## Data Availability

All data are publicly available. The original contributions presented in the study are included in the article/**Supplementary Material**. Further inquiries can be directed to the corresponding authors.

## Supplementary material

Supplementary Figure S1 Mean Serum Cotinine Concentration by Time Period. Estimates of the geometric mean serum cotinine concentration and its 95% CI are plotted for each of 3 time periods (1988-1991, 1999-2004, and 2011-2012), along with the best-fitting trend line. These estimates are based on the 13,288 NHANES participants aged ≥12 years with data on both ANA and serum cotinine. Any concentration below the limit of detection (LOD) was replaced by an imputed value equal to LOD/ $\sqrt{2}$ . The horizontal axis is linear in time, defined as the number of years between the midpoints of the participant’s period and the first period, and the vertical axis is logarithmic in serum cotinine concentration (ng/mL).
