# Supplementary material for: Decreased cigarette smoking may partially explain the increased prevalence of antinuclear antibodies in the United States
Source: Front Immunol. 2025 Aug 27;16:1537043. doi: 10.3389/fimmu.2025.1537043 (PMC12420209; doi:10.3389/fimmu.2025.1537043)
Supplement: Supplementary Figure S1 — Mean Serum Cotinine Concentration by Time Period. Estimates of the geometric mean serum cotinine concentration and its 95% CI are plotted for each of 3 time periods (1988-1991, 1999-2004, and 2011-2012), along with the best-fitting trend line. These estimates are based on the 13,288 NHANES participants aged ≥12 years with data on both ANA and serum cotinine. Any concentration below the limit of detection (LOD) was replaced by an imputed value equal to LOD/ 2 . The horizontal axis is linear in time, defined as the number of years between the midpoints of the participant’s period and the first period, and the vertical axis is logarithmic in serum cotinine concentration (ng/mL). [file DataSheet1.pdf]

Supplementary Material for "Decreased cigarette smoking may partially explain the increased prevalence of antinuclear antibodies in the United States"

Supplemental Figure S1. Mean Serum Cotinine Concentration by Time Period. Estimates of the geometric mean serum cotinine concentration and its 95% CI are plotted for each of 3 time periods (1988-1991, 1999-2004, and 2011-2012), along with the best-fitting trend line. These estimates are based on the 13,288 NHANES participants aged ≥12 years with data on both ANA and serum cotinine. Any concentration below the limit of detection (LOD) was replaced by an imputed value equal to LOD/$\sqrt{2}$. The horizontal axis is linear in time, defined as the number of years between the midpoints of the participant's period and the first period, and the vertical axis is logarithmic in serum cotinine concentration (ng/mL).

Supplemental Table S1. Number of NHANES Participants with Data on ANA, Smoking Exposure, and Smoking History by Time Period and Age Range.

Supplemental Table S2. Sample Size and Geometric Mean Serum Cotinine Concentration (ng/mL) by Time Period, Smoking History, and Age Range.

Supplemental Table S3. Estimated Prevalence of Smoking in NHANES by Time Period and Age Range.

Supplemental Table S4. Covariate-Adjusted Estimates of the Smoking Prevalence Odds Ratios for Time Period by Smoker Type and Age Range.

Supplemental Table S5. Covariate-Adjusted Assessments of ANA Time Trends (Without Any Stratification).

Supplemental Table S6. Covariate-Adjusted Assessments of ANA Time Trends by Smoking Exposure After Adding a BMI Main Effect and a BMI-by-Age Interaction to the Base Model.

Supplemental Figure S1. Mean Serum Cotinine Concentration by Time Period.

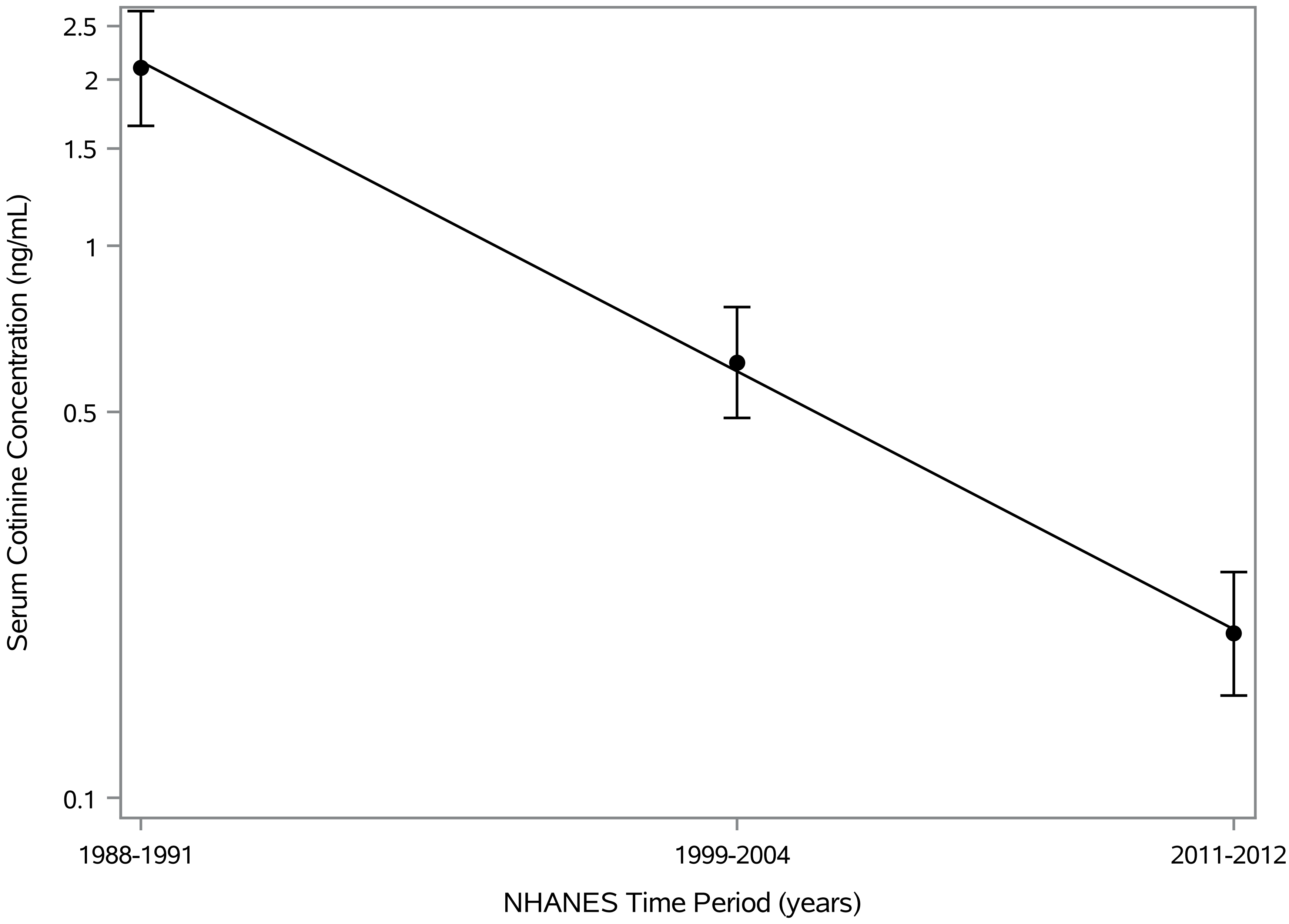

Supplemental Table S1. Number of NHANES Participants with Data on ANA, Smoking Exposure, and Smoking History by Time Period and Age Range.

Data Subset	Period 1: 1988-1991	Period 2: 1999-2004	Period 3: 2011-2012	All Periods Combined
		All Participants: Ages 12 Years or Older		
ANA	4,727	4,527	4,265	13,519
ANA and Smoking Exposure	4,525	4,499	4,264	13,288
ANA and Smoking History	4,332	4,451	3,495	12,278
ANA, Smoking Exposure, and Smoking History	4,145	4,424	3,494	12,063
		Adult Participants: Ages 20 years or Older		
ANA	4,051	3,429	3,498	10,978
ANA and Smoking Exposure	3,869	3,408	3,497	10,774
ANA and Smoking History	4,051	3,427	3,495	10,973
ANA, Smoking Exposure, and Smoking History	3,869	3,406	3,494	10,769

Abbreviations: ANA = antinuclear antibodies; NHANES = National Health and Nutrition Examination Survey.

Supplemental Table S2. Sample Size and Geometric Mean Serum Cotinine Concentration (ng/mL) by Time Period, Smoking History, and Age Range.

	Self-Reported Smoking History Category							
	Never Smoker		Former Smoker		Current Smoker		Overall	
Time Period	N	Mean (95% CI)	N	Mean (95% CI)	N	Mean (95% CI)	N	Mean (95% CI)
All Participants: Ages 12 Years or Older								
Period 1: 1988-1991	1,988	0.27 (0.22-0.33)	1,009	0.59 (0.44-0.78)	1,148	158.5 (139.5-180.2)	4,525	2.11 (1.66-2.67)
Period 2: 1999-2004	2,440	0.09 (0.07-0.10)	1,059	0.18 (0.14-0.24)	925	104.2 (91.4-118.8)	4,499	0.58 (0.46-0.72)
Period 3: 2011-2012	1,988	0.04 (0.03-0.04)	822	0.08 (0.06-0.10)	684	139.3 (118.3-164.0)	4,264	0.20 (0.15-0.25)
All Periods Combined	6,416	0.08 (0.08-0.09)	2,890	0.19 (0.15-0.23)	2,757	131.6 (121.3-142.9)	13,288	0.57 (0.49-0.67)
Adult Participants: Ages 20 years or Older								
Period 1: 1988-1991	1,786	0.26 (0.22-0.30)	991	0.58 (0.44-0.77)	1,092	158.9 (139.5-180.9)	3,869	2.32 (1.80-2.99)
Period 2: 1999-2004	1,713	0.08 (0.06-0.09)	935	0.17 (0.13-0.23)	758	125.9 (111.6-142.1)	3,408	0.60 (0.47-0.75)
Period 3: 2011-2012	1,988	0.04 (0.03-0.04)	822	0.08 (0.06-0.10)	684	139.3 (118.3-164.0)	3,497	0.22 (0.17-0.28)
All Periods Combined	5,487	0.08 (0.07-0.09)	2,748	0.18 (0.15-0.23)	2,534	141.3 (130.6-153.0)	10,774	0.62 (0.53-0.72)

Abbreviations: CI = confidence interval; N = total number of participants (sample size); NHANES = National Health and Nutrition Examination Survey.

Each geometric mean was calculated by exponentiating the arithmetic mean of the log-transformed serum cotinine concentrations in the appropriate subgroup. Adjustments were made for the survey design variables (sampling weights, strata, and clusters). Smoking history categories (Never, Former, and Current) were based on self-reports from questionnaire data. The sample size in the last column is larger than the sum of those for the 3 smoking history subgroups because some participants did not have data on smoking history but still contributed to the overall period-specific means. Smoking history data were available for participants of ages 17+ years in Period 1, ages 12+ years in Period 2, and ages 20+ years in Period 3. The top half of the table includes all participants (ages 12+ years) and the bottom half restricts participants to a common age range of 20+ years.

Supplemental Table S3. Estimated Prevalence of Smoking in NHANES by Time Period and Age Range.

Time Period	Estimated Prevalence of Smoker Type as a Percentage (95% CI)	
	Active Smoker (cotinine-based)	Current Smoker (self-reported)
	All Participants: Ages 12 Years or Older	
Period 1: 1988-1991	33.1 (30.8-35.5)	30.5 (27.8-33.4)
Period 2: 1999-2004	27.5 (25.3-29.9)	24.3 (22.4-26.2)
Period 3: 2011-2012	21.2 (18.6-24.1)	19.5 (17.2-22.0)
	Adult Participants: Ages 20 years or Older	
Period 1: 1988-1991	34.9 (32.4-37.6)	30.7 (28.0-33.6)
Period 2: 1999-2004	28.9 (26.5-31.5)	24.7 (22.7-26.8)
Period 3: 2011-2012	23.0 (20.4-25.8)	19.5 (17.2-22.0)

Abbreviations: CI = confidence interval; NHANES = National Health and Nutrition Examination Survey.

Statistical Model: Separately for each age range, smoking prevalence was estimated under a logistic regression model for the binary indicator of active smoking (serum cotinine concentration >10 ng/mL) or current smoking (self-reported in questionnaire). The model adjusted for the survey design variables (sampling weights, strata, and clusters) and a single categorical covariate for time period.

Supplemental Table S4. Covariate-Adjusted Estimates of the Smoking Prevalence Odds Ratios for Time Period by Smoker Type and Age Range.

Time Period	Smoking Prevalence Odds Ratio for Time Period (95% CI)	
	Active Smoker (cotinine-based)	Current Smoker (self-reported)
	All Participants: Ages 12 Years or Older	
Period 1: 1988-1991	1.00 (reference)	1.00 (reference)
Period 2: 1999-2004	0.80 (0.68-0.95)	0.76 (0.64-0.90)
Period 3: 2011-2012	0.59 (0.47-0.72)	0.58 (0.47-0.73)
	Adult Participants: Ages 20 years or Older	
Period 1: 1988-1991	1.00 (reference)	1.00 (reference)
Period 2: 1999-2004	0.80 (0.67-0.95)	0.76 (0.64-0.91)
Period 3: 2011-2012	0.61 (0.49-0.74)	0.59 (0.47-0.73)

Abbreviations: CI = confidence interval; NHANES = National Health and Nutrition Examination Survey.

Statistical Model: Separately for each age range, smoking prevalence was estimated under a logistic regression model for the binary indicator of active smoking (serum cotinine concentration >10 ng/mL) or current smoking (self-reported in questionnaire). The model adjusted for the survey design variables (sampling weights, strata, and clusters) and categorical covariates for sex, age, and race/ethnicity. The model also included a categorical covariate for time period, which allowed estimates of the smoking prevalence odds ratio for each period relative to the first period.

Supplemental Table S5. Covariate-Adjusted Assessments of ANA Time Trends (Without Any Stratification).

Model Covariates	N+	N	ANA Prevalence Odds Ratio for Time Period (95% CI) Period 1: 1988-1991	Period 2: 1999-2004	Period 3: 2011-2012	Time Trend p-value
Base Model: Main Effects for Sex, Age, Race/Ethnicity, and Period or Time						
Categorical Age (8 groups)	1,857	13,519	1.00 (reference)	1.02 (0.84-1.24)	1.49 (1.23-1.82)	0.0001
Substitute an Alternative Age Variable in the Base Model						
Categorical Age (3 groups)	1,857	13,519	1.00 (reference)	1.02 (0.84-1.24)	1.49 (1.23-1.81)	<0.0001
Quantitative Age	1,857	13,519	1.00 (reference)	1.02 (0.84-1.23)	1.50 (1.23-181)	<0.0001
Restricted Cubic Spline in Age	1,857	13,519	1.00 (reference)	1.02 (0.84-1.24)	1.50 (1.23-1.83)	<0.0001
Add a Main Effect for One Other Factor to the Base Model						
Base + Smoking Exposure	1,816	13,288	1.00 (reference)	1.01 (0.83-1.24)	1.46 (1.20-1.77)	0.0001
Base + BMI	1,824	13,326	1.00 (reference)	1.02 (0.84-1.24)	1.48 (1.23-1.79)	<0.0001
Base + Alcohol Intake (ages 20+ years)	1,374	9,496	1.00 (reference)	0.92 (0.73-1.16)	1.54 (1.23-193)	0.0004
Base + PIR	1,689	12,348	1.00 (reference)	0.98 (0.80-1.21)	1.51 (1.24-1.84)	<0.0001
Base + Education	1,793	13,132	1.00 (reference)	1.03 (0.85-1.25)	1.50 (1.23-1.84)	0.0001
Add Main Effects for BMI and One Other Factor						
Base + BMI + Smoking Exposure	1,784	13,100	1.00 (reference)	1.02 (0.83-1.25)	1.45 (1.20-1.76)	0.0001
Base + BMI + Alcohol Intake (ages 20+ years)	1,356	9,403	1.00 (reference)	0.91 (0.73-1.15)	1.52 (1.22-1.88)	0.0003
Base + BMI + PIR	1,662	12,176	1.00 (reference)	0.99 (0.81-1.23)	1.49 (1.23-1.81)	<0.0001
Base + BMI + Education	1,761	12,950	1.00 (reference)	1.03 (0.85-1.25)	1.48 (1.23-1.79)	<0.0001
Add a BMI Main Effect and a BMI Interaction with One Other Factor (and a Main Effect for the Other Factor if Not in the Base Model)						
Base + BMI + BMI*Sex	1,824	13,326	1.00 (reference)	1.02 (0.85-1.24)	1.48 (1.23-1.79)	<0.0001
Base + BMI + BMI*Age (3 groups)	1,824	13,326	1.00 (reference)	1.02 (0.84-1.24)	1.48 (1.23-1.78)	<0.0001
Base + BMI + BMI*Race/Ethnicity	1,824	13,326	1.00 (reference)	1.02 (0.85-1.24)	1.48 (1.23-1.79)	<0.0001
Base + BMI + SmokExpo + BMI*SmokExpo	1,784	13,100	1.00 (reference)	1.03 (0.83-1.26)	1.45 (1.20-1.77)	<0.0001
Base + BMI + Alc + BMI*Alc (ages 20+ years)	1,356	9,403	1.00 (reference)	0.91 (0.73-1.15)	1.52 (1.23-1.89)	0.0003
Base + BMI + PIR + BMI*PIR	1,662	12,176	1.00 (reference)	0.99 (0.81-1.23)	1.49 (1.23-1.81)	<0.0001
Base + BMI + Education + BMI*Education	1,761	12,950	1.00 (reference)	1.03 (0.85-1.24)	1.47 (1.22-1.77)	<0.0001

Abbreviations: Alc = alcohol intake; ANA = antinuclear antibodies; BMI = body mass index; CI = confidence interval; N = total number of participants (sample size); N+ = number of ANA-positive participants; PIR = poverty income ratio; SmokExpo = smoking exposure.

Statistical Models: ANA time trend assessments were based on two logistic regression models for ANA positivity (yes/no). Each model adjusted for the survey design variables (sampling weights, strata, and clusters) and categorical covariates for sex, age, and race/ethnicity. One model added a categorical covariate for time period, which allowed estimates of the ANA prevalence odds ratio for each period relative to the first period. The other model added a continuous covariate for time, as measured by the number of years between period midpoints relative to the first period, and produced a p-value from a t-test to assess an ANA time trend. Both models added certain main effects in many cases and also added a BMI interaction in some cases.

Variable Definitions: The base model included categorical variables for sex, age (12-19, 20-29, ..., 70-79, 80+ years), and race/ethnicity (non-Hispanic White, non-Hispanic Black, Mexican American, Other). Alternative age variables included categorical age (12-19, 20-49, 50+ years), quantitative age, and a restricted cubic spline in age. The smoking exposure categories were based on serum cotinine concentrations (<=0.05 ng/mL, >0.05 to 10 ng/mL, >10 ng/mL). The BMI categories were <25 kg/m2, 25 to <30 kg/m2, and >=30 kg/m2 for ages 20+ years, but were determined by the year 2000 US Centers for Disease Control and Prevention growth chart percentiles (<85, 85 to <95, >=95) for ages 12-19 years. Alcohol intake data were only available for ages 20+ years and the categories were based on the number of drinks consumed in the past year (<12 total, 1 to 3 per week, >3 per week). The PIR categories were at/above poverty (>=1) and below poverty (<1). The education categories were less than high school, high school, and more than high school.

Supplemental Table S6. Covariate-Adjusted Assessments of ANA Time Trends by Smoking Exposure After Adding a BMI Main Effect and a BMI-by-Age Interaction to the Base Model.

Smoking Exposure	ANA Prevalence Odds Ratio for Time Period (95% CI)			Time Trend p-value
	Period 1: 1988-1991	Period 2: 1999-2004	Period 3: 2011-2012	
None	1.00 (reference)	0.68 (0.43-1.05)	0.95 (0.60-1.50)	0.2647
Passive	1.00 (reference)	1.31 (0.99-1.74)	1.80 (1.38-2.35)	<0.0001
Active	1.00 (reference)	0.80 (0.53-1.23)	1.37 (0.93-2.02)	0.1432
Total	1.00 (reference)	1.02 (0.85-1.24)	1.49 (1.23-1.80)	<0.0001

Abbreviations: ANA = antinuclear antibodies; CI = confidence interval.

Statistical Models: ANA time trend assessments were based on two logistic regression models for ANA positivity (yes/no). Each model adjusted for the survey design variables (sampling weights, strata, and clusters) and categorical covariates for sex, age, race/ethnicity, and BMI, plus a BMI-by-age interaction. One model added a categorical covariate for time period, which allowed estimates of the ANA prevalence odds ratio for each period relative to the first period. The other model added a continuous covariate for time, as measured by the number of years between period midpoints relative to the first period, and produced a p-value from a t-test to assess an ANA time trend. Both models were applied initially to all participants with data on ANA regardless of data on smoking exposure (Total) and then within subgroups with data on both ANA and smoking exposure (None, Passive, and Active). The smoking exposure categories were based on serum cotinine concentrations (None, <=0.05 ng/mL; Passive, >0.05 to 10 ng/mL; and Active, >10 ng/mL).
